# Deep learning assessment compared to radiologist reporting for metastatic spinal cord compression on CT

**DOI:** 10.3389/fonc.2023.1151073

**Published:** 2023-05-04

**Authors:** James Thomas Patrick Decourcy Hallinan, Lei Zhu, Wenqiao Zhang, Shuliang Ge, Faimee Erwan Muhamat Nor, Han Yang Ong, Sterling Ellis Eide, Amanda J. L. Cheng, Tricia Kuah, Desmond Shi Wei Lim, Xi Zhen Low, Kuan Yuen Yeong, Mona I. AlMuhaish, Ahmed Mohamed Alsooreti, Nesaretnam Barr Kumarakulasinghe, Ee Chin Teo, Qai Ven Yap, Yiong Huak Chan, Shuxun Lin, Jiong Hao Tan, Naresh Kumar, Balamurugan A. Vellayappan, Beng Chin Ooi, Swee Tian Quek, Andrew Makmur

**Affiliations:** ^1^Department of Diagnostic Imaging, National University Hospital, Singapore, Singapore; ^2^Department of Diagnostic Radiology, Yong Loo Lin School of Medicine, National University of Singapore, Singapore, Singapore; ^3^Department of Computer Science, School of Computing, National University of Singapore, Singapore, Singapore; ^4^Department of Radiology, Ng Teng Fong General Hospital, Singapore, Singapore; ^5^Department of Radiology, Imam Abdulrahman Bin Faisal University, Dammam, Saudi Arabia; ^6^Department of Diagnostic Imaging, Salmaniya Medical Complex, Manama, Bahrain; ^7^National University Cancer Institute, National University Hospital, Singapore, Singapore; ^8^Biostatistics Unit, Yong Loo Lin School of Medicine, Singapore, Singapore; ^9^Division of Spine Surgery, Department of Orthopaedic Surgery, Ng Teng Fong General Hospital, Singapore, Singapore; ^10^University Spine Centre, Department of Orthopaedic Surgery, National University Health System, Singapore, Singapore; ^11^Department of Radiation Oncology, National University Cancer Institute Singapore, National University Hospital, Singapore, Singapore

**Keywords:** metastatic spinal cord compression (MSCC), Epidural spinal cord compression, metastatic epidural spinal cord compression (MESCC), spinal metastatic disease, deep learning, artificial intelligence, CT, MRI

## Abstract

**Introduction:**

Metastatic spinal cord compression (MSCC) is a disastrous complication of advanced malignancy. A deep learning (DL) algorithm for MSCC classification on CT could expedite timely diagnosis. In this study, we externally test a DL algorithm for MSCC classification on CT and compare with radiologist assessment.

**Methods:**

Retrospective collection of CT and corresponding MRI from patients with suspected MSCC was conducted from September 2007 to September 2020. Exclusion criteria were scans with instrumentation, no intravenous contrast, motion artefacts and non-thoracic coverage. Internal CT dataset split was 84% for training/validation and 16% for testing. An external test set was also utilised. Internal training/validation sets were labelled by radiologists with spine imaging specialization (6 and 11-years post-board certification) and were used to further develop a DL algorithm for MSCC classification. The spine imaging specialist (11-years expertise) labelled the test sets (reference standard). For evaluation of DL algorithm performance, internal and external test data were independently reviewed by four radiologists: two spine specialists (Rad1 and Rad2, 7 and 5-years post-board certification, respectively) and two oncological imaging specialists (Rad3 and Rad4, 3 and 5-years post-board certification, respectively). DL model performance was also compared against the CT report issued by the radiologist in a real clinical setting. Inter-rater agreement (Gwet’s kappa) and sensitivity/specificity/AUCs were calculated.

**Results:**

Overall, 420 CT scans were evaluated (225 patients, mean age=60 ± 11.9[SD]); 354(84%) CTs for training/validation and 66(16%) CTs for internal testing. The DL algorithm showed high inter-rater agreement for three-class MSCC grading with kappas of 0.872 (p<0.001) and 0.844 (p<0.001) on internal and external testing, respectively. On internal testing DL algorithm inter-rater agreement (κ=0.872) was superior to Rad 2 (κ=0.795) and Rad 3 (κ=0.724) (both p<0.001). DL algorithm kappa of 0.844 on external testing was superior to Rad 3 (κ=0.721) (p<0.001). CT report classification of high-grade MSCC disease was poor with only slight inter-rater agreement (κ=0.027) and low sensitivity (44.0), relative to the DL algorithm with almost-perfect inter-rater agreement (κ=0.813) and high sensitivity (94.0) (p<0.001).

**Conclusion:**

Deep learning algorithm for metastatic spinal cord compression on CT showed superior performance to the CT report issued by experienced radiologists and could aid earlier diagnosis.

## 1 Introduction

Vertebral metastases are common and affect approximately 40% of patients with cancer (1). Vertebral metastases can be complicated by back pain, pathological fractures, and metastatic spinal cord compression (MSCC) (2). MSCC occurs in around 10% of patients with spinal metastases and is considered an oncological emergency. Permanent disability in MSCC can be averted if there is earlier diagnosis using cross-sectional imaging, allowing appropriate therapy to be planned and initiated (3, 4).

The earliest symptom of MSCC is most commonly pain, which precedes neurological dysfunction including altered sensations, reduced ambulation, and eventual paralysis (5). Unfortunately, if a patient with MSCC is non-ambulatory at presentation, regaining mobility following treatment is unlikely (3, 4). Clinical diagnosis of MSCC can be delayed as the earliest signs and symptoms may be subtle, especially regarding back pain which may be difficult to separate from pre-existing degeneration and/or masked by opioid analgesics. Patients with early stage MSCC can also be asymptomatic, which makes it challenging to detect the disease at an earlier stage when less invasive treatment such as radiotherapy can be initiated (2, 6).

MRI is the imaging modality of choice for MSCC as it provides detailed assessment of the vertebrae, spinal cord and surrounding CSF (4, 6, 7). This allows for assessment of the degree of MSCC and is vital for treatment planning including stereotactic body radiotherapy (SBRT). MSCC is most frequently graded using the Spine Oncology Study Group classification, developed by Mark Bilsky et al. in 2010 (7). The scale consists of six groupings, which can be further divided into two key subgroups for treatment planning— Low-grade MSCC (0/1a/1b) is typically considered for radiotherapy (including external beam or SBRT), or high-grade MSCC with impending or frank spinal cord compression (1c/2/3) is typically steered towards primary surgical decompression and subsequent radiotherapy (8, 9).

CT myelography is another accurate modality for diagnosis of MSCC, but it is usually reserved for patients with contraindications to MRI (e.g., pacemakers) as the procedure is invasive requiring injection of contrast into the thecal sac. Staging CT scans with intravenous contrast are performed at frequent intervals in oncology patients to assess response to treatment and detect new metastatic lesions (10). These staging CT scans have the potential to provide an earlier diagnosis of MSCC in patients with subtle or absent symptoms, and further clinical and MRI assessment can then be undertaken. Crocker et al. (2011) assessed the use of contrast-enhanced CT for detection of MSCC, and reported high specificity of 92%, and sensitivity of 89% when compared against MRI (11).

Deep learning (DL) assistance for classifying MSCC on staging CT could improve the clinical workflow for patients, allowing for early clinical assessment and high-resolution MRI for treatment planning, especially if SBRT or surgical intervention is indicated. Artificial intelligence applications in spine MRI and CT have shown potential clinical utility, including semi-automated grading of spinal canal stenosis (12, 13), and assessment of skeletal metastatic burden on PET/CT (14). Most recently, Hallinan et al. (2022) trained and tested preliminary CT deep learning algorithms for classification of MSCC (15). The DL algorithms showed good interrater variability compared to an expert radiologist (reference standard) with kappa values between 0.873 to 0.911 (p<0.001). The study only assessed performance on an internal test set, and did not assess the generalisability of the model at an external institution or the accuracy of the DL algorithm relative to the formal radiology report for each staging CT study.

The aim of this study is to further develop and assess the performance of a deep learning algorithm for MSCC detection and grading on CT scans. These automated DL tools could expedite timely diagnosis of MSCC and improve selection of treatment including stereotactic radiotherapy or surgical decompression. The reference standard MSCC gradings on CT will be prepared using matched MR images interpreted by radiologists with expertise in spine imaging. The performance of the DL algorithm will be compared against the pre-existing staging CT radiology reports (generated in a real clinical setting) and focused assessment for MSCC by radiologists experienced in spine or oncological body imaging. In addition, the performance and generalizability of the DL algorithm will be assessed on a dataset from an external institution.

## 2 Materials and methods

This retrospective study was conducted at both internal and external healthcare institutions. It was approved by the local institutional review board (National Healthcare Group (NHG), Singapore; protocol code NHG DSRB Ref: 2020/00835 dated 17 September 2021), and all analysis was carried out in accordance with relevant guidelines and regulations. The requirement for informed consent was waived by the local institutional review board owing to the retrospective nature of this study and low risk to participants.

### 2.1 Dataset preparation

Retrospective, manual retrieval of CT staging examinations and corresponding MRI spines from patients with suspected MSCC was conducted over a thirteen-year period from September 2007 through to September 2020 at NUH, Singapore. Inclusion criteria were adults (≥18 years-of-age) with corresponding MRI and CT studies performed ≤ 60 days of each other. **Tables 1**, **2** indicate the imaging specifications for the MRI and CT examinations, which were performed using a wide variety of platforms. A heterogeneous set of imaging data for training and testing is useful to create a generalizable DL algorithm. Exclusion criteria were CT or MRI studies with instrumentation, no intravenous CT contrast, extensive motion artefacts/poor image quality and studies covering the upper cervical and lumbosacral regions. For the MRI assessment, transverse T2-weighted sequences were examined.

**Table 1:** CT scanner details.

Parameter	CT1	CT2	CT3	CT4	CT5	CT6*
Number of slices	4	64	256	384	512	128
Pitch	1.5	1.2	0.984	0.8	0.531	0.55
Slice thickness (mm)	5	5	3	3	3	3
Collimation (mm)	4 x 1	32 x 0.6	128 x 0.625	192 x 0.6	256 x 0.625	128 x 0.6
kV	120	120	100	100	100-120	100-140
Reference mAs	180	200	250	200	200	170-200
Rotation time (s)	0.5	0.5	0.5	0.5	0.5	0.28

kV, Kilovoltage; mAs, Milliampere-seconds, *CT scanner present at the external institution (NTFGH, Singapore). All the five other CT scanners were situated at the internal institution (NUH, Singapore). All patient scans were performed in a craniocaudal direction whilst the patient was lying down. Contrast volume for all CT scanners = 70-100ml depending on patient size, at a rate of 1.2-1.5 mL/s.

**Table 2:** MRI scanner details for transverse T2-weighted sequences (reference standard).

Parameter	MRI 1 and 2	MRI 3	MRI 4	MRI 5	MRI 6*
Tesla (T)	1.5	1.5	3	3	1.5
TR (msec)	3500	4000	5300	5300	2000
TE (msec)	80	90	100	100	87
Slice thickness (mm)	5	5	5	5	4
Gap (mm)	6	6	6	6	10
Field of view (mm^2^)	200 x 200	160 x 160	200 x 200	160 x 160	180 x 180
Matrix	512 x 512	320 x 320	512 x 512	640 x 640	384 x 384

TR, repetition time; TE, echo time; *MRI scanner present at the external institution (NTFGH, Singapore). All the five other MRI scanners were situated at the internal institution (NUH, Singapore). All patient scans were performed whilst the patient was lying down with application of a body coil.

The internal imaging data was obtained at the Internal Institution (NUH, Singapore) and was divided at random into 84% and 16% for the deep learning algorithm training/validation and held-out test data sets, respectively. This division of datasets for training, validation and testing of DL algorithms is acceptable (16).

A dataset of staging CT and corresponding MRI spine examination from patients with suspected MSCC were also retrieved for external testing from Ng Teng Fong General Hospital (NTFGH), Singapore (imaging parameters documented in **Tables 1**, **2**). Inclusion and exclusion criteria for the external dataset were the same as for the primary institutional datasets. The imaging studies were retrieved over a five-year period between September 2015 through to September 2020. The external dataset was for testing the DL algorithm with no further training conducted.

### 2.2 Dataset labelling

Internal training image sets were labelled by radiologists with specialization in musculoskeletal/spine imaging (SRad1; 11 years post board certification) and neuroimaging (SRad2; 6 years post board certification). Each labeller assessed over 180 CT and corresponding MRI thoracic spine studies independently. Using an open-source data labelling tool (LabelImg1.8.6: https://github.com/heartexlabs/labelImg), boxes were outlined along the CT images of the thoracic spinal canal to highlight the area of interest for the DL algorithm training. The thoracic region encompassed a transverse imaging volume from C7 through to L3 (approximate location of the conus medullaris). The transverse CT images are extracted from the original DICOM file in three standardized CT window widths and levels (W/L in Hounsfield units): Bone (1500/300), Abdomen (400/50), and Spine (200/100). These are then used to train both the object detection and classification DL algorithms without further pre-processing. The rationale for using multiple windows is that they are used by clinicians to provide more accurate analysis for MSCC, including optimal assessment of the bony margins and enhancing epidural soft tissue.

**Figure 1:**
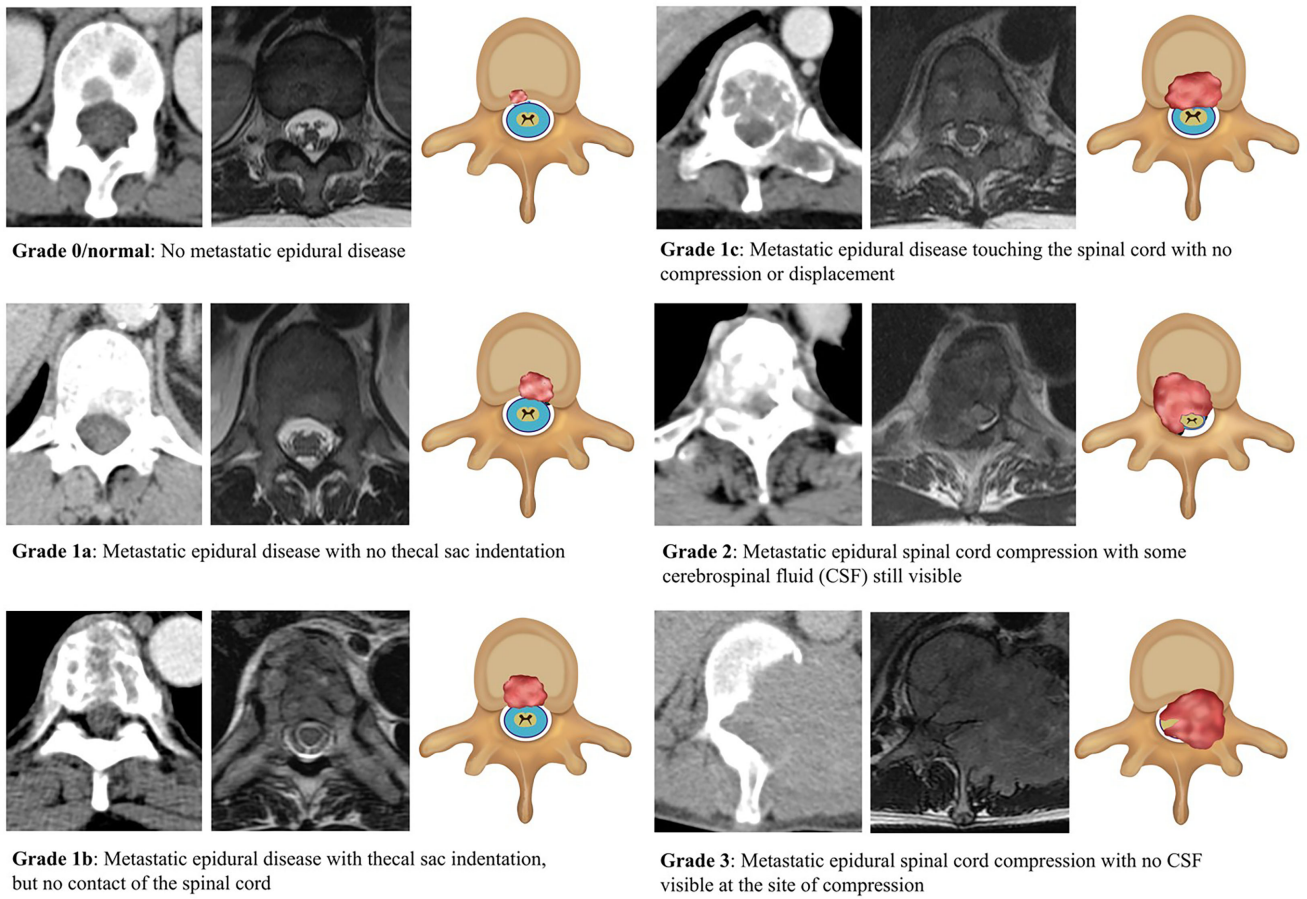
Classification/grading of metastatic spinal cord compression (MSCC) using the Bilsky grading scale with corresponding CT, MRI, and graphic illustrations (right to left). MSCC is highlighted by the red-shaded areas. Axial CT was performed in the portal-venous phase and matching axial MRI images were T2-weighted, which are best for showing the cerebrospinal fluid (blue), spinal cord (yellow), and surrounding structures.

The labellers classified each axial CT image using the Bilsky MSCC classification in conjunction with the axial T2W MRI studies. For this study the Bilsky grading was divided into normal (0/normal/no epidural disease), low (1a/1b) and high-grade (1c/2/3) disease, which is useful for treatment planning. A visual guide to the Bilsky scale using diagrams, MRI and CT images was provided to all the radiologists (**Figure 1**). Marked narrowing of the spinal canal due to degenerative disk osteophyte disease, and/or ossification of the yellow ligament were highlighted by the specialist radiologists and excluded from training and testing of the DL algorithms (17, 18).

Both the held-out internal and external CT test data sets were assessed using the MSCC grading scale by the specialist radiologist (SRad1) in conjunction with the MR images. These gradings served as the internal and external reference standards. The performance of the developed DL algorithm was compared against another group of radiologists on the internal and external institution test sets using inter-rater agreement. The radiologists labelled the test sets independently and included two specialist musculoskeletal radiologists (Rad1 and Rad2, 7- and 5-years post board certification, respectively), along with two radiologists with experience in oncological CT evaluation (Rad3 and Rad4, 3 and 5 years post board certification, respectively). All readers had access to the visual Bilsky scale and were provided with ten CT and corresponding MRI examples of normal, low or high-grade MSCC classification prior to assessing the test sets. None of the readers had access to the DL algorithm predictions, MR images or reference standard gradings during the CT test set assessments.

### 2.3 Original CT radiologist reports

The DL algorithm performance was also compared to the original CT reports of the internal test set cases generated at the time of scanning. Each staging CT study is issued with a formal written report by a board-certified radiologist. These reports should detail information concerning the primary tumour, secondary metastatic disease, treatment response, and any complications including MSCC. Each report was examined by the experienced radiologists (SRad1 and SRad2) to assess for documentation on the presence of MSCC (yes/no), site (vertebral level), and predicted Bilsky grade (normal, low, or high-grade).

### 2.4 Deep learning algorithm development

We developed a consecutive region of interest (ROI) detector and classification/grading deep learning pipeline following the study by Hallinan and Zhu et al., 2022 (15). First, we build Faster R-CNN (19) combined with ResNet50 (20) as its backbone network architecture, which consists of 50 convolutional layers with ReLU (21) activation function for non-linearity. We fine-tune the model on our newly collected training data with the ROI annotations as the supervision with a learning rate of 0.0003 for 200k steps. We use stochastic gradient descent (SGD) (22) with a momentum of 0.9 for optimization and a weight decay of 0.0005 for regularization following (19). The fine-tuned model is used for ROI detection. Second, the combined window learning (CWL) (15) method with average fusion is adopted for ROI classification with multi-window information due to its robustness to missing input window information and reduced model size. Specifically, we build the CWL model with a convolutional prototypical network (23) using ResNeXt50 (24) as its backbone network architecture. The ResNeXt50 network consists of 50 convolutional layers with ReLU activation function and specially designed aggregated transformations. We chose ResNeXt50 rather than its deeper (e.g., ResNeXt101) or shallower counterparts (e.g., ResNeXt29) in our study due to its good fit to our dataset size to avoid overfitting or underfitting. As CT scans with different window information display different characteristics, we apply window-specific batch normalization layers (25) to capture the window-specific information and share the remaining weights of the model to capture the common information.

**Figure 2:**
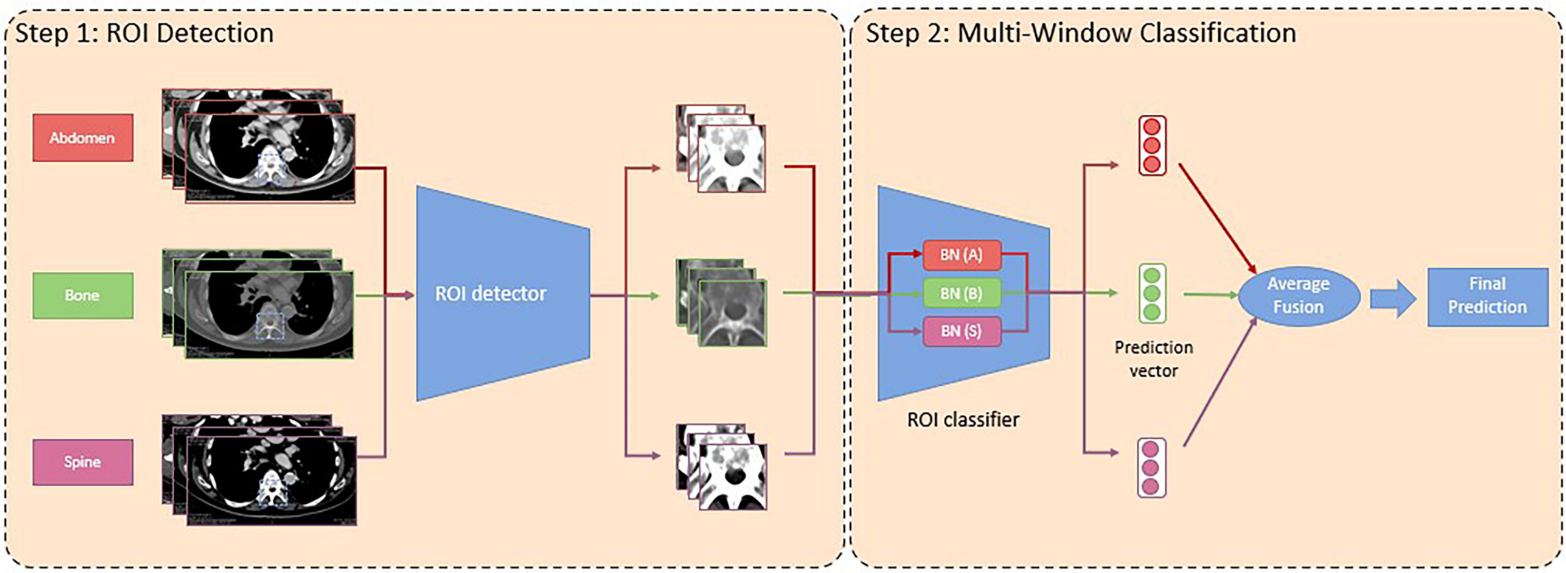
Graphical summary of the deep learning (DL) algorithms, which formed a pipeline. The deep learning pipeline consumes input data (images) with information from three different CT windows, namely abdomen, bone, and spine. In the first portion, a region of interest (ROI) detector is used to identify the ROIs for all images with different window information. In the second step, a ROI classifier with a window-specific batch normalization layer is applied to each input image with different window information to obtain their prediction vectors. Later, the prediction vectors are element-wisely added and averaged as the final prediction. BN, Batch Normalization.

During each training iteration, our model receives a batch of inputs which consists of data samples with different window information of equal size and apply the corresponding batch normalization layers on them for backpropagation. We use SGD with a learning rate of 0.001 for optimization and a weight decay of 0.0005 for regularization. We train our model for 200 epochs. For inference, our model receives input with multi-window information and outputs the average prediction probability based on the input information from each window as the final prediction probability. The hyper-parameters of our deep learning algorithm are optimized using a separate, held-out validation data set. **Figure 2** presents a graphical summary of the deep learning algorithms, which form a pipeline. An ablation study is also provided in **Table 3** to evaluate how much performance benefits arise from the framework and how much are coming from the advanced backbone.

**Table 3:** Ablation study on different backbone network architectures and learning frameworks for three-grade MSCC classification.

Backbone Framework	ResNet50	ResNext50	Increment
Plain	78.55	79.80	+1.24
CWL	80.52	82.50	+1.99
Increment	+1.96	+2.70	–

CWL, Combined Window Learning. Numbers in the table are the average class accuracy results evaluated on the internal test set for each combination of backbone network architecture and learning framework to indicate their classification performance.

Our deep learning pipeline was created using the Apache SINGA (26) platform. We also use MLCask (27), a system which facilitates efficient management of several versions of the deep learning algorithms/pipeline. During each training iteration, the batch size is set to be 72 with inputs of equal size from the three different windows on a NVIDIA GeForce GTX 1080p GPU for the training of the CWL model. The code of the deep learning algorithms is adopted from SpineAI@NUHS-NUS (available for review at https://github.com/NUHS-NUS-SpineAI/SpineAI-Bilsky-Grading-CT).

### 2.5 Statistical analysis

Analysis of the data was undertaken using Stata software version 17 (Stata Statistical Software: Release 17. College Station, TX: StataCorp LLC), with statistical significance determined at 2-sided p<0.05. Predicting that kappas of approximately 0.9 are to be generated with a 95% confidence interval (CI) width of 0.1, at least 138 or more CT and matched MRI studies are required. Continuous variables were presented as means with standard deviations, while categorical variables were shown as numbers (%). Inter-rater agreement using three-grade (0/normal, low or high-grade) and two-grade (0/normal or low-grade versus high-grade, and 0/normal versus low or high-grade) MSCC classifications were assessed using Gwet’s kappa to take into account the high percentage of grade 0/normal images (28). AUCs, sensitivities, and specificities were presented for all two-grade MSCC groupings and furnished with 95% CIs. For comparison with the radiology reports, the performance of the DL algorithm and radiologists were assessed for detection of a high-grade MSCC lesion on each CT study (i.e., two-grade 0/normal or low-grade versus high-grade classification) against the reference standard.

Strength of agreement for Gwet’s kappa statistics: Almost-perfect (1-0.81), substantial (0.8-0.61), moderate (0.6-0.41), fair (0.4-0.21), slight (0.2-0), and poor (<0) (29).

## 3 Results

### 3.1 Patient demographics within the datasets

Dataset analysis over the thirteen-year period identified 225 patients with 513 CT scans and matched MRI spines for analysis. Overall, out of the 513 CT studies, 93 were excluded from analysis as they were either studies covering only the upper cervical and lumbosacral regions (49/93, 52.7%), no intravenous contrast was administered (32/93, 34.4%), spinal instrumentation was *in situ* (6/93, 6.5%), there was a greater than two-month (60-day) interval between the staging CT scan and spine MRI (3/93, 3.2%), or extensive motion artefacts/poor quality images were present (3/93, 3.2%). In total 420 CT scans from 225 patients were available for deep learning algorithm internal training/validation and testing.

The mean age of all 225 patients was 60 ± 11.9(SD) with a range from 18 to 93 years old. The patient group was comprised of slightly more males (117/225, 52.0%) than females (108/225, 48.0%) with lung and breast malignancies the most common (104/225 patients, 46.2%). There was a heterogeneous spread of MSCC sites along the thoracic spine, with the majority of disease between T11 through to L3 (85/225, 37.8%). **Table 4** illustrates the patient demographics, types of cancer, and thoracic MSCC locations.

**Table 4:** Patient Details and Clinical data for the test datasets.

Characteristics	Internal training/validation set (n=183)	Internal test set (n=42)	External Test set (n=32)
Age (years)*	60 ± 12.3 (18-93)	62± 10 (40-82)	60 ± 13 (19-85)
Women	89 (51.4)	19 (45.2)	12 (37.5)
Men	94 (48.6)	23 (54.8)	20 (62.5)
Cancer Subtype			
Lung	45 (24.6)	10 (23.8)	13 (40.6)
Breast	36 (19.7)	13 (31.0)	3 (9.4)
Renal cell carcinoma	17 (9.3)	3 (7.1)	3 (9.4)
Colon	16 (8.7)	3 (7.1)	4 (12.5)
Prostate	13 (7.1)	1 (2.4)	1 (3.1)
Multiple Myeloma	11 (6.0)	3 (7.1)	0 (0)
Hepatocellular carcinoma	9 (4.9)	2 (4.8)	0 (0)
Nasopharyngeal carcinoma	9 (4.9)	0 (0)	1 (3.1)
Others	27 (14.8)	7 (16.7)	7 (21.9)
MSCC location			
C7-T2	19 (10.4)	1 (2.4)	6 (18.8)
T3-T10	49 (26.8)	15 (35.7)	15 (46.9)
T11-L3	68 (37.2)	17 (40.5)	8 (25)
Diffuse thoracic^#^	31 (16.9)	8 (19.0)	3 (9.4)
No epidural disease	16 (8.7)	1 (2.4)	0 (0)
No. of staging CT thoracic studies	354/420 (84)	66/420 (16)	43

MSCC, Malignant spinal cord compression. *The values provided are mean in years of age ± SD (range indicated) for numerical variables, along with n (%) for categorical variables. ^#^Two or more sites of MSCC along the thoracic spine.

**Figure 3:**
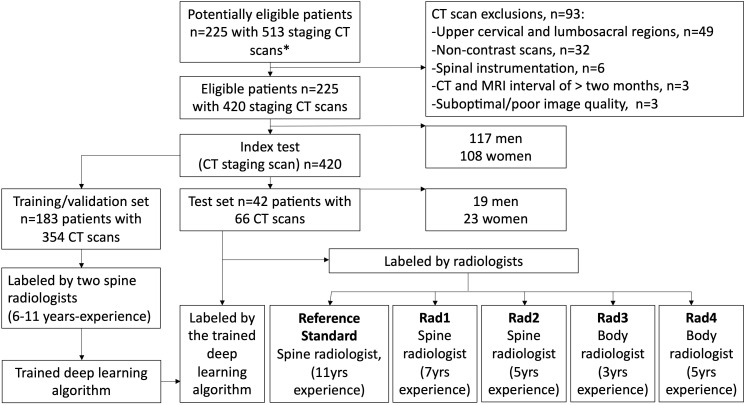
Flow chart of the deep learning algorithm training and testing study design. The algorithm performance was compared with a spine radiologist (reference standard with access to corresponding MRI scans) and four radiologists (blinded to the MRI spines). *All CT scans had matched MRI spine scans.

The internal dataset totaling 420 CT scans was randomly separated into 354 (84%) scans for training/validation, and 66 (16%) scans for internal testing with no patient overlap between the groups (flow chart in **Figure 3**). For the external dataset, 43 staging CT studies covering the thoracic spine from 32 patients with suspected MSCC were retrieved for testing (**Table 4**). Overall, mean age for the 32 external patients was 60 ± 13 (SD) with a range from 19 to 85 years old. As for the internal data set, the majority of the patient group were male (20/32, 62.5%), and lung cancer represented the most frequent primary malignancy (13/32, 40.6%).

### 3.2 Reference standard CT MSCC gradings

The total number of CT images and matched MSCC gradings for the internal training, validation and test sets, along with the external institution test set are provided in **Table 5**. For the internal training, validation and test sets, any grade of MSCC accounted for 2560/12488 (20.5%), 604/3133 (19.3%), and 638/3331 (19.2%), regions of interest respectively. In the external test set a similar distribution was noted with 282/1558 (18.1%) regions of interest for both low and high-grade MSCC.

**Table 5:** MSCC classification reference standards for the internal and external CT data sets.

MSCC classification	Internal Training Set	Internal Validation Set	Internal Test Set	External Test Set
Normal/grade 0	9928 (79.5)	2529 (80.7)	2693 (80.8)	1276 (81.9)
Low-grade 1a/1b	1412 (11.3)	303 (9.7)	351 (10.5)	105 (6.7)
High-grade 1c/2/3	1148 (9.2)	301 (9.6)	287 (8.6)	177 (11.4)
**Total**	12488	3133	3331	1558

Values are n (%) and represent the number of axial CT images and corresponding MSCC grade. MSCC, Metastatic Spinal Cord Compression.

### 3.3 Three-grade MSCC classification

For three-grade MSCC classification, the DL algorithm showed almost perfect inter-rater agreement on the internal test data (κ =0.872, 95% CI 0.859-0.886), which was superior to Rad 2 a spine imaging specialist (κ = 0.795, 95% CI 0.778-0.811) and Rad 3 a body imaging specialist (κ = 0.724, 95% CI 0.705-0.743) (both p<0.001) (**Table 6**). The DL algorithm performance showed no evidence of a significant difference compared to an experienced spine radiologist (Rad 1, κ = 0.883, 95% CI 0.871-0.896) and a body imaging specialist (Rad 4, κ = 0.870, 95% CI 0.856-0.883) (p>0.05 for both).

**Table 6:** Inter-rater agreement for three-grade MSCC classification.

	Internal Test Set		External Test Set	
Reader	Kappa (95% CI)	P value	Kappa (95% CI)	P value
DL algorithm	0.872 (0.859- 0.886)	<0.001	0.844 (0.822- 0.865)	<0.001
Rad 1	0.883 (0.871- 0.896)	<0.001	0.902 (0.885- 0.918)	<0.001
Rad 2	0.795 (0.778- 0.811)	<0.001	0.862 (0.842- 0.882)	<0.001
Rad 3	0.724 (0.705- 0.743)	<0.001	0.721 (0.693- 0.749)	<0.001
Rad 4	0.870 (0.856- 0.883)	<0.001	0.901 (0.884- 0.918)	<0.001

DL, Deep Learning.

On the external institution dataset, the DL algorithm displayed similar almost perfect inter-rater agreement for three-grade MSCC classification with a kappa of 0.844 (95% CI 0.822-0.865), which was significantly superior to Rad 3 a body imaging specialist (κ =0.721, 95% CI 0.693-0.749) (p<0.001) (**Table 6**).

### 3.4 Low or high-grade MSCC classification

The DL algorithm and radiologist performance was assessed for detection of any grade of MSCC. For this classification on the internal dataset, the DL algorithm displayed almost perfect inter-rater agreement (κ = 0.910, 95% CI 0.897-0.922), which was superior to Rad 2 a spine specialist (κ = 0.793, 95% CI 0.773-0.813), Rad 3 (κ = 0.678, 95% CI 0.654-0.703) and Rad 4 (κ = 0.871, 95% CI 0.857-0.886) (all p<0.001) (**Table 7**). For the same classification on the external dataset, the DL algorithm also displayed almost perfect inter-rater agreement (κ = 0.909, 95% CI 0.890-0.927), which was superior Rad 3 (κ = 0.686, 95% CI 0.650-0.722) (p<0.001).

**Table 7:** Inter-rater agreement for classification of normal versus any grade of MSCC.

	Internal Test Set		External Test Set	
Reader	Kappa (95% CI)	P value	Kappa (95% CI)	P value
DL algorithm	0.910 (0.897- 0.922)	<0.001	0.909 (0.890- 0.927)	<0.001
Rad 1	0.904 (0.891- 0.917)	<0.001	0.914 (0.896- 0.931)	<0.001
Rad 2	0.793 (0.773- 0.813)	<0.001	0.882 (0.861- 0.903)	<0.001
Rad 3	0.678 (0.654- 0.703)	<0.001	0.686 (0.650- 0.722)	<0.001
Rad 4	0.871 (0.857- 0.886)	<0.001	0.928 (0.912- 0.944)	<0.001

Any grade of MSCC classification is defined as two-grade normal/none versus low or-high grade disease. DL, Deep Learning.

For detection of any grade of MSCC on the internal dataset the DL algorithm displayed high sensitivity (87.13, 95% CI 84.28-89.63), specificity (95.43, 95% CI 94.58-96.19), and AUC (0.913, 95% CI 0.899-0.926). The DL algorithm showed similar performance on the external dataset with high sensitivity (96.45, 95% CI 93.58-98.29), specificity (93.26, 95% CI 91.74-94.57), and AUC (0.949, 95% CI 0.936-0.961) (**Table 8**).

**Table 8:** Sensitivity, specificity and AUC for classification of normal versus any grade of MSCC.

	Internal Test Set			External Test Set		
Reader	Sensitivity (95% CI)	Specificity (95% CI)	AUC (95% CI)	Sensitivity (95% CI)	Specificity (95% CI)	AUC (95% CI)
DL Algorithm	87.13 (84.28-89.63)	95.43 (94.58-96.19)	0.913 (0.899-0.926)	96.45 (93.58-98.29)	93.26 (91.74-94.57)	0.949 (0.936-0.961)
Rad 1	67.82 (64.04-71.43)	99.00 (98.55-99.34)	0.834 (0.816-0.852)	75.18 (69.71-80.11)	97.88 (96.94-98.60)	0.865 (0.840-0.891)
Rad 2	81.79 (78.57-84.71)	87.60 (86.30-88.82)	0.847 (0.831-0.863)	91.13 (87.19-94.18)	92.24 (90.63-93.65)	0.917 (0.899-0.935)
Rad 3	89.64 (87.01-91.90)	78.32 (76.72-79.87)	0.840 (0.826-0.854)	97.16 (94.49-98.77)	77.43 (75.03-79.70)	0.873 (0.858-0.888)
Rad 4	51.49 (47.53-55.44)	99.52 (99.18-99.74)	0.755 (0.736-0.775)	80.85 (75.77-85.27)	97.96 (97.03-98.66)	0.894 (0.871-0.917)

### 3.5 High-grade MSCC classification

The DL algorithm and radiologist performance was also assessed for detection of high-grade MSCC. For this classification on the internal dataset, the DL algorithm displayed almost perfect inter-rater agreement (κ = 0.942, 95% CI 0.933-0.951), which showed no evidence of a significant difference compared to Rad 2 (a spine imaging specialist) who had the lowest kappa of 0.941 (95% CI 0.931-0.950) (p>0.05) (**Table 9**). For the same classification on the external dataset, the DL algorithm also displayed almost perfect inter-rater agreement (κ = 0.950, 95% CI 0.937-0.962), which was superior to Rad 2 who had the lowest kappa of 0.929 (95% CI 0.914-0.945) (p=0.042).

**Table 9:** Inter-rater agreement for classification of normal/low versus high-grade MSCC.

	Internal Test Set		External Test Set	
Reader	Kappa (95% CI)	P value	Kappa (95% CI)	P value
DL algorithm	0.942 (0.933- 0.951)	<0.001	0.950 (0.937- 0.962)	<0.001
Rad 1	0.957 (0.950- 0.965)	<0.001	0.942 (0.928- 0.955)	<0.001
Rad 2	0.941 (0.931- 0.950)	<0.001	0.929 (0.914- 0.945)	<0.001
Rad 3	0.968 (0.962- 0.975)	<0.001	0.957 (0.945- 0.968)	<0.001
Rad 4	0.949 (0.941- 0.958)	<0.001	0.937 (0.922- 0.951)	<0.001

High-grade MSCC classification is defined as two-grade normal/none or low-grade versus high-grade disease. DL, Deep Learning.

For detection of high-grade MSCC on the internal dataset the DL algorithm showed high sensitivity (93.38, 95% CI 89.85-95.97), specificity (95.47, 95% CI 94.67-96.18), and AUC (0.944, 95% CI 0.929-0.959). The DL algorithm showed similar performance on the external dataset with high sensitivity (96.61, 95% CI 92.77-98.75), specificity (96.02, 95% CI 94.85-96.99), and AUC (0.963, 95% CI 0.949-0.977) (**Table 10**).

**Table 10:** Sensitivity, specificity and AUC for classification of normal/low versus high-grade MSCC.

	Internal Test Set			External Test Set		
Reader	Sensitivity (95% CI)	Specificity(95% CI)	AUC (95% CI)	Sensitivity(95% CI)	Specificity(95% CI)	AUC (95% CI)
DL Algorithm	93.38 (89.85-95.97)	95.47 (94.67-96.18)	0.944 (0.929-0.959)	96.61 (92.77-98.75)	96.02 (94.85-96.99)	0.963 (0.949-0.977)
Rad 1	60.28 (54.36-65.98)	99.67 (99.40-99.84)	0.800 (0.771-0.828)	71.19 (63.91-77.73)	98.33 (97.51-98.94)	0.848 (0.814-0.881)
Rad 2	81.53 (76.55-85.85)	96.35 (95.63-96.99)	0.889 (0.867-0.912)	92.66 (87.77-96.03)	94.79 (93.48-95.90)	0.937 (0.917-0.957)
Rad 3	78.75 (73.55-83.33)	99.05 (98.63-99.36)	0.889 (0.865-0.913)	94.92 (90.57-97.65)	96.81 (95.75-97.68)	0.959 (0.942-0.976)
Rad 4	63.07 (57.20-68.66)	98.72 (98.25-99.09)	0.809 (0.781-0.837)	88.70 (83.09-92.96)	95.87 (94.69-96.86)	0.923 (0.899-0.947)

The DL algorithm, radiologists and original radiology reports were also compared for detection of a high-grade MSCC lesion on each CT scan against the reference standard (**Table 11**). Out of the 66 CT scans analysed (approximately 50 axial images per scan), 50/60 (83.3%) scans had at least one high-grade MSCC lesion and 16/60 (26.7%) had no high-grade MSCC lesion. All radiologists and the DL algorithm showed superior inter-rater agreement compared to the original reports (p<0.001). For example, the DL algorithm demonstrated almost perfect inter-rater agreement with kappa of 0.813 (95% CI 0.678-0.949) and AUC of 0.814 (95% CI 0.692-0.936), which were superior to the original radiology report kappa of only 0.027 (95% CI -0.232-0.286) and AUC of 0.564 (95% CI 0.427-0.700) (p<0.001 and p=0.007, respectively). All radiologists and the DL algorithm showed superior sensitivities compared to the original radiology reports, e.g., Rads 1 and 4 had the lowest sensitivity of 76.0 (95% CI 61.83- 86.94), p=0.001 and the DL algorithm had sensitivity of 94.0 (95% CI 83.45-98.75), compared to the original report sensitivity of only 44.0 (95% CI 29.99-58.75) (p<0.001).

**Table 11:** Classification of high-grade MSCC per CT scan.

Reader	Kappa(95% CI)	P-value	Sensitivity(95% CI)	Specificity (95% CI)	AUC (95% CI)
DL Algorithm	0.813 (0.678- 0.949)	<0.001	94.0 (83.45- 98.75)	68.75 (41.34- 88.98)	0.814 (0.692- 0.936)
Rad 1	0.673 (0.489- 0.857)	<0.001	76.0 (61.83- 86.94)	100.00 (79.41- 100.00)	0.880 (0.820- 0.940)
Rad 2	0.813 (0.678- 0.949)	<0.001	94.0 (83.45- 98.75)	68.75 (41.34- 88.98)	0.814 (0.692- 0.936)
Rad 3	0.849 (0.725- 0.974)	<0.001	90.0 (78.19- 96.67)	93.75 (69.77- 99.84)	0.919 (0.844- 0.993)
Rad 4	0.603 (0.401- 0.804)	<0.001	76.0 (61.83- 86.94)	81.25 (54.35- 95.95)	0.786 (0.671- 0.902)
Original Report	0.027 (-0.232-0.286)	0.836	44.00 (29.99- 58.75)	68.75 (41.34- 88.98)	0.564 (0.427- 0.700)

High-grade MSCC classification is defined as two-grade normal/none or low-grade versus high-grade disease. Original report was the formal written evaluation issued by the radiologist at the time of the CT scan. DL, Deep Learning.

## 4 Discussion

MSCC is a serious complication of advanced cancer, and MRI is the current gold standard imaging test to confirm the diagnosis and plan therapy (30). However, MRI is an expensive procedure and is reserved for confirming a clinical suspicion of MSCC typically due to new back pain and/or neurological symptoms. Staging CT scans are commonly performed for cancer diagnosis and treatment follow-up and represent a window of opportunity for earlier diagnosis of MSCC, especially when the clinical signs/symptoms are unclear. In this study, we further trained and externally tested an existing DL algorithm for the detection of any grade of MSCC on staging CT studies. Upon testing, the DL algorithm showed almost-perfect inter-rater agreement for three-class MSCC grading with kappas of 0.872 (p<0.001) and 0.844 (p<0.001) on the internal and external test data sets, respectively. On the internal test set the DL algorithm inter-rater agreement (κ = 0.872) was superior to Rad 2, a spine imaging specialist (κ = 0.795) and Rad 3 a body imaging specialist (κ =0.724) (both p<0.001). The DL algorithm kappa of 0.844 on the external test set was also superior to Rad 3 (κ =0.721) (p<0.001).

Analysis of the staging CT reports (issued by the radiologist at the time of the study) revealed that the classification of high-grade MSCC disease was poor with only slight inter-rater agreement (κ = 0.027) and low sensitivity (44.0). In comparison, the DL model showed superior performance (p<0.001) with almost-perfect inter-rater agreement (κ = 0.813) and high sensitivity (94.0). This classification is especially important as patients with high-grade MSCC are at risk of progression to irreversible spinal cord injury and require urgent work-up with MRI for treatment planning.

DL tools for spine conditions on cross-sectional imaging include MRI assessment of spinal canal stenosis (12, 31), radiotherapy planning for vertebral tumours to exclude organs at risk including the spinal cord (32), and for differentiating benign versus malignant spinal tumours (33). DL algorithms for automated Bilsky grading of MSCC have also been explored on MRI (34) and CT (15). The preliminary DL algorithms for MSCC grading on CT showed kappas of 0.873-0.911 (p<0.001) on an internal test set and were used as the basis for this study (15). The DL algorithm underwent further training on an expanded internal training set and showed sustained performance on a larger internal test set with kappa of 0.872 (p<0.001). More importantly, in this study, the DL algorithm for CT MSCC classification showed sustained almost-perfect inter-rater agreement on an external dataset (κ = 0.844, p<0.001) suggesting the model could be generalizable across institutions. This latter step can be overlooked in clinical DL algorithms and is important to prevent site/selection bias and overfitting to the internal training data set (35).

An automated DL algorithm for MSCC classification could optimize the clinical workflow of patients with suspected MSCC. Firstly, at the time of the CT scan, the DL tool could triage cases for early reporting of MSCC by a radiologist through an automated text alert system. This is feasible as DL tools have been successfully deployed for stroke triage including the detection of acute intracranial hemorrhage and large vessel occlusion on CT studies (36). Secondly, when reporting the study, the DL MSCC tool could also highlight key axial CT images with MSCC for the radiologist to review and document in the report. This is referred to as DL assisted reporting, which has recently been shown to improve the productivity and consistency of radiologists when reporting lumbar spinal stenosis on MRI and CT of the thorax (31, 37). Finally, the key images selected by the DL model and radiologist could be provided to the requesting clinician and circulated to experts in spinal metastasis management including the radiation oncology and spine surgery teams. Overall, improvement in the detection and communication of MSCC could result in earlier diagnosis of the condition and improved patient outcomes, including preservation of ambulation.

Our retrospective study has several limitations. First, we used isolated axial CT images for the Bilsky grading of MSCC. The addition of multiplanar sagittal and coronal images to train a three-dimensional convolutional neural network could further improve the DL algorithm performance. Second, an assessment of the time delays between the CT and MRI could not be completed due to incomplete clinical records at the time of the CT scans. Most patients attending for an outpatient CT study would not be assessed by the clinical team and screened for MSCC (e.g., back pain and neurological signs). Third, patients with CT studies performed within 60 days of an MRI spine were included in this study. This time window was used to balance the risk of disease progression between the studies with the amount of training data available for the DL algorithm. Fourth, the use of focused radiologist assessment of MSCC on CT is not representative of real clinical practice, which involves assessment of all organs (lungs, liver, etc.), osseous structures and soft tissues. However, focused MSCC assessment on CT represented a more rigorous comparison (i.e., best possible radiologist performance) for the developed DL algorithm. Finally, the potential clinical impact of the DL algorithm was assessed against a reference standard focusing on imaging alone. In practice, it must be emphasized that the decision for surgery and/or radiotherapy combines both imaging and clinical findings, including the type of cancer (radiosensitivity), prognosis and comorbidities, and presence of neurological compromise.

In conclusion, we further developed, internally tested, and externally validated a deep learning (DL) algorithm for the grading of metastatic spinal cord compression using routine staging CT scans. The DL algorithm had almost-perfect inter-rater agreement (kappas of 0.872 and 0.844 on internal and external testing, respectively) similar or superior to focussed radiologist assessment for the determination of normal, low, or high-grade MSCC. For detection of high-grade MSCC per CT scan, the DL algorithm showed superior inter-rater agreement with kappa of 0.813 compared to the original radiology report kappa of only 0.027 (p<0.001). Future work would involve deploying the CT MSCC classification model onto the radiology reporting platform at several institutions to assess the generalizability and prospective diagnostic performance.

## Data availability statement

The raw data supporting the conclusions of this article will be made available by the authors, without undue reservation.

## Ethics statement

The studies involving human participants were reviewed and approved by the local institutional review board (National Healthcare Group (NHG), Singapore; protocol code NHG DSRB Ref: 2020/00835). Written informed consent for participation was not required for this study in accordance with the national legislation and the institutional requirements.

## Author contributions

Conception, methodology, data curation, supervision, visualization, and writing: JH, LZ, WZ, SG, FN, HO, SE, AC, TK, DL, XL, KY, MA, AA, NBK, ET, QY, YC, SL, JT, NK, BV, BO, SQ and AM. Investigation and project administration: JH, LZ, WZ, SG, FN, HO, SE, AC, TK, DL, XL, KY, ET, SL, JT, NK, BV, BO, SQ and AM. Resources and software: JH, LZ, WZ, SG, KY, QY, YC, JT, NK, BO, SQ and AM. Formal analysis and validation: JH, LZ, WZ, SG, QY, YC, SL, JT, NK, BV, BO, SQ and AM. All authors contributed to the article and approved the submitted version.
